# Relationship of serum adiponectin and resistin levels with the severity of liver fibrosis in patients with chronic hepatitis B

**DOI:** 10.5937/jomb0-33793

**Published:** 2022-04-08

**Authors:** Nerma Čustović, Senija Rašić

**Affiliations:** 1 University of Sarajevo, Clinical Center, Clinic for Gastroenterohepatology, Sarajevo, Bosnia and Herzegovina; 2 University of Sarajevo, Faculty of Medicine, Department of Internal Medicine, Sarajevo, Bosnia and Herzegovina

**Keywords:** adipocytokines, APRI, FIB-4, hepatitis B, adipocitokini, APRI, FIB-4, hepatitis B

## Abstract

**Background:**

Recent research has closely linked adipocytokines to liver inflammation and fibrosis progression in patients with non-alcoholic liver disease. This study aimed to determine the relationship of serum adiponectin and resistin levels with the severity of liver fibrosis in patients with chronic hepatitis B (CHB), depending on the duration of antiviral therapy.

**Methods:**

The cross-sectional study included 75 patients with CHB divided into two groups: the T1 group (undergoing antiviral therapy for up to 2 years) and the T2 group (undergoing antiviral therapy over 2 years). The control group consisted of 40 healthy people. Serum concentrations of adiponectin and resistin were estimated with the ELISA method, while the degree of liver fibrosis was determined using FIB-4 and APRI score.

**Results:**

There were no statistically significant differences in the mean serum adiponectin levels in relation to the duration of antiviral therapy. Higher values of serum resistin concentration were confirmed in patients of the T1 group compared to healthy controls (p=0.001) and to the T2 group (p=0.031). The mean level of serum resistin concentration was significantly higher in the group of patients with a higher FIB-4 score (9.12±3.39 vs 5.58±3.36 ng/mL, p=0.001) and higher APRI score (17.45±3.96 ng/mL vs 4.82±1.11 ng/mL, p=0.001). A positive correlation was found between serum resistin levels and the degree of liver fibrosis (p<0.001). There was no significant difference between mean serum adiponectin levels according to the values of FIB-4 and APRI scores.

**Conclusions:**

Progression of liver fibrosis estimated by FIB4 and APRI scores as well as the length of antiviral treatment had a significant effect on serum resistin values in CHB patients on antiviral therapy.

## Abstract (sr)

**Uvod:**

Novija istraživanja su usko povezala adipocitokine sa progresijom upale i fibroze jetre kod bolesnika koji imaju bolesti jetre koje nisu povezane sa konzumiranjem alkohola. Cilj ovog istraživanja bio je da se utvrdi odnos serumskog nivoa adiponektina i rezistina sa težinom fibroze jetre kod pacijenata sa hroničnim hepatitisom B (CHB), u zavisnosti od trajanja antivirusne terapije.

**Metode:**

Studija preseka je obuhvatila 75 pacijenata sa HHB koji su bili podeljeni u dve grupe: T1 grupa (na antivirusnoj terapiji do 2 godine) i T2 grupa (na antivirusnoj terapiji preko 2 godine). Kontrolnu grupu je činilo 40 zdravih osoba. Serumske koncentracije adiponektina i rezistina su procenjene uz pomoć ELISA metode, dok je stepen fibroze jetre određen pomoću FIB-4 i APRI skora.

**Rezultati:**

Nije bilo statistički značajnih razlika u srednjim nivoima adiponektina u serumu u odnosu na trajanje antivirusne terapije. Veće vrednosti koncentracije rezistina u serumu su potvrđene kod pacijenata iz T1 grupe u poređenju sa zdravim pacijentima iz kontrolne grupe (p = 0,001) i pacijentima iz grupe T2 (p=0,031). Prosečan nivo koncentracije rezistina u serumu bio je značajno veći u grupi pacijenata sa većim skorom FIB-4 (9,12 ± 3,39 naspram 5,58 ± 3,36 ng/mL, p = 0,001) i većim skorom APRI (17,45 ± 3,96 ng/mL naspram 4,82 ± 1,11 ng/mL, p=0,001). Utvrđena je pozitivna korelacija između serumskog nivoa rezistina i stepena fibroze jetre (p < 0,001). Nije bilo značajne razlike između srednjih nivoa adiponektina u serumu prema vrednostima FIB-4 i APRI skoru.

**Zaključak:**

Napredovanje fibroze jetre procenjeno rezultatima FIB-4 i APRI, kao i du'ina antivirusnog lečenja su imali značajan uticaj na vrednosti rezistina u serumu kod pacijenata sa hroničnim hepatitisom B na antivirusnoj terapiji.

## Introduction

Adipose tissue is an important endocrine organ, which regulates a wide variety of physiological functions through the secretion of adipocytokines [1] [2]. Adipose tissue and the liver cooperatively regulate energy homeostasis. A recent study by Chang et al. [3] confirmed that non-alcoholic fatty liver disease and viral hepatitis cause specific alterations in adipocytokine profiles. Proinflammatory effects of resistin and anti-inflammatory effects of adiponectin have been shown in various metabolic and inflammatory diseases (atherosclerosis, diabetes mellitus, fatty liver disease, viral hepatitis), including patients with chronic hepatitis B [4] [5]. Research by Hsu et al. [6] has shown that serum adipocytokine levels independently correlate with liver fibrosis stages in patients with chronic hepatitis B (CHB), which classifies them as prognostic markers.

Improved non-invasive tests, Fibrosis-4 score (FIB-4) and Aspartate Aminotransferase-to-Platelet Ratio Index (APRI score) during antiviral therapy are a reflection of treatment-regression in liver histopathology [7] [8]. Considering that CHB requires long-term antiviral therapy, monitoring the effect of therapy on the regression of liver fibrosis using non-invasive methods and parameters is of great importance, especially nowadays when the rate of hepatocellular carcinoma is extremely high, presenting the third leading cause of cancer death among the cancers of digestive system worldwide [9].

The role of adipocytokines in non-alcoholic liver disease has been extensively investigated [10] [11], but their role in patients with CHB is not sufficiently clarified through research to date. Therefore, this study aimed to determine the importance of serum adiponectin and resistin levels as prognostic markers and indicators of the liver fibrosis stages in patients with CHB, depending on the duration of antiviral therapy.

## Materials and Methods

### Study population

Seventy-five patients (42 men and 33 women, mean age 52.5 years) with a confirmed diagnosis of CHB, treated with antiviral therapy by nucleoside analog tenofovir, were included in the cross-sectional study. The clinical criteria for the diagnosis of CHB were: positive hepatitis B surface antigen (HBsAg) for at least 6 months, increased alanine aminotransferase (ALAT) and detectable serum hepatitis B virus (HBV) DNA by PCR test. The study was conducted at the Clinic for Gastroenterohepatology, Clinical Center of the University of Sarajevo, from January 2020 to December 2020.

All patients were divided into two groups: the T1 group included 37 patients on antiviral therapy for up to 2 years, while the T2 group included 38 patients on antiviral therapy for more than 2 years. Antiviral therapy with tenofovir was administered at a dose of 245 mg per os once daily for a long time, according to defined guidelines for treating viral hepatitis B [12]. The study did not include patients with radiologic evidence of hepatocellular carcinoma, hepatitis C virus coinfection, autoimmune liver disease, liver cirrhosis, diabetes mellitus and previous history of alcohol consumption, patients with a BMI>25 as well as the patients with hepatosplenomegaly, ascites, peripheral edema, jaundice, and signs of liver cirrhosis.

The control group (CG) consisted of 40 healthy persons (22 male and 18 female), mean age 52.0 years, recruited from subjects who underwent preventive examination at the Counseling Centre for Gastroenterohepatology, at the Clinical Center of the University of Sarajevo. They had no clinical and laboratory signs of liver and metabolic disease. Additionally, patients and the control group who took hepatotoxic or fatty liver-inducing medicines (estrogen, amiodarone, methotrexate, tamoxifen) in the past three months were not included in the study.

All respondents gave informed consent to participate in the study. The study protocol was approved by the local Ethical Committee (No: 03-02-3083/2019). The study was conducted according to ethical standards of medical research and the Declaration of Helsinki.

### Methods

A detailed history was taken from patients treated for chronic hepatitis B on the day of enrollment in the study and during a follow-up examination. Abdominal examination was performed in all participants in the study as an essential part of all routine physical examinations. Body height was measured without shoes on a height measuring scale, while the body weight was measured using a digital scale. Body mass index (BMI) was calculated as the ratio of body weight in kilograms (kg) to the square of body height in meters (m^2^).

The presence of liver fibrosis and the degree of its severity was determined using the FIB-4 score, ranging from <1.45 (negative predictive value for advanced fibrosis) to >3.25 (positive predictive value for advanced fibrosis) and also APRI score ranging from <0.5 (negative predictive value for advanced fibrosis) to >1.45 (positive predictive value for advanced fibrosis). The values of FIB-4 and APRI scores were calculated using formulas, which included serum aspartate aminotransferase (AST) and alanine aminotransferase (ALT) values, as well as platelet count and age values [13] [14].

### Blood sampling and measurement

Blood samples were taken on the day of inclusion in the study from the cubital vein of fasting patients in non-heparinized tubes and then centrifuged at 4000 rpm for 10 minutes. All separated serum samples for determination of adiponectin and resistin concentration were stored at -70°C until laboratory analysis, while serum levels of AST and ALT were determined on the day of the blood sampling as well as the platelet count from the full blood.

Serum adiponectin concentration was assessed using a commercial serum adiponectin level kit (Demeditec Diagnostics GmbH, Kiel, Germany), while serum resistin concentration was assessed using a commercial resistin concentration kit (Demeditec Diagnostics GmbH, Kiel, Germany). Both biomarkers were analyzed by immunoenzymometric assays (ELISA sandwich-assays) with two specific antibodies. The adiponectin and resistin in the samples first binded to the antibody coated on the microtiter plate. In the next step, the second specific anti-adiponectinantibody and the anti-resistin-antibody binded to the immobilized adiponectin and resistin. The second antibody was biotinylated and administered in a mixture with the peroxidase-enzyme conjugate. The enzymatic reaction led to the appearance of colour in proportion to the concentration of adipocytokine levels of the samples. After antibody conjugation, stop solution was added to the plate, followed by incubation at room temperature in a plate shaker with a rotation frequency of 350 rpm. The results were read spectrophotometrically at a wavelength of 450 nm on a plate reader BioTek ELX50 (Winooski, Vermont, USA). The measured adiponectin concentration was expressed in micrograms per millilitre (mg/mL), while the resistin concentration was expressed in nanograms per millilitre (ng/mL).

Serum levels of liver transaminases (AST, ALT) were determined by enzyme analysis on COBAS 6000 (Roche, Basel, Switzerland), while the platelet count was determined on the counter CELL-DYN Ruby (Abbott, Illinois, United States). In addition, serum HBV DNA levels were determined with a commercially available quantitative polymerase chain reaction (PCR) assay (Amplicor HBV Monitor; Roche Molecular Diagnostics Systems, Branchburg, N.J., USA).

### Statistical analysis

The statistical analysis was performed using the Statistical Package for the Social Science (SPSS) software, version 22 for Windows (SPSS Inc, Chicago, Illinois, SAD). The normality of data distribution was determined by the Kolmogorov-Smirnov test. Variables with normal distribution were presented as mean ± standard deviation (SD) and compared using the t-test for independent samples. ANOVA test was used for statistical evaluation of variables of more than two groups. The post-hoc Scheffe test was used to uncover specific differences between three or more group means when an analysis of variance (ANOVA) was significant. A univariate two-way ANOVA test was performed to examine the effects of independent variables on the dependent variable and their interaction effects. The Spearman correlation coefficient was used to analyze the relationships of the monitored variables. The significance level was set at *p*<0.05.

## Results

### Clinical characteristics

There was no statistically significant difference between the study groups concerning the mean age of patients and gender, while a significant difference was confirmed in the degree of liver fibrosis between two groups of patients with different duration of disease treatment. Patients on antiviral treatment up to 2 years had a higher degree of liver fibrosis based on values of APRI and FIB-4 score in comparison with patients who have been on therapy for more than 2 years (p<0.001). The basic characteristics of the study groups are presented in Table 1.

**Table 1:** Basic characteristics of patients with chronic viral hepatitis and control group CG – control group; T1 – patients on antiviral therapy for up to 2 years; T2 – patients on antiviral therapy for more than 2 years

Variables	CG<br> (n= 40)	T1 group<br> (n=37)	T2 group<br> (n=38)	p
Age (mean)	52.0±3.0	54.0±2.0	51.0±5.0	0.541
Male (n, %)<br> Female (n, %)	22/55%<br> 18/45%	20/54%<br> 17/46%	22/57%<br> 16/43%	0.443<br> 0.608
BMI (kg/m2)	22.76±1.91	22.72±1.97	22.79±1.89	0.885
Treatment length (years)		1.8±0.1	4.3±0.2	0.001
FIB-4 score		1.84±0.72	1.28±0.71	0.001
APRI score		0.75±0.44	0.39±0.25	0.001

### Serum adiponectin and resistin levels

Serum adiponectin values were significantly lower in the group of patients on antiviral therapy longer than 2 years compared to the control group (13.35±7.82 vs 17.67±7.16 mg/mL, p=0.047), while there were no statistically significant differences between the control group and group of patients on antiviral therapy up to 2 years (p=0.50), as well as between two groups of patient with duration of antiviral treatment up to and over 2 years (15.44±8.06 vs 13.35±7.82 mg/mL p=0.448), Table 2.

**Table 2:** The values of adiponectin and resistin serum concentration in treated patients with chronic viral hepatitis B and control group Data are presented as mean ± SD.<br>CG – control group; T1 – CHB patients on antiviral therapy for up to 2 years; T2 – CHB patients on antiviral therapy for more than 2 years; ^*^p<0.05 comparison of serum adiponectin level in the CG and T2 groups; ^⁑^p<0.01 – serum resistin level between T1 and CG group; ^┴^p<0.05 – serum resistin level between T1 and T2 group; ^₸^p<0.01 – serum resistin level between T2 and CG group

Variables	CG	T1 group	T2 group	p
Adiponectin (mg/mL)	17.67±7.16	15.44±8.06	13.35±7.82*	0.049
Resistin (ng/mL)	4.47±1.61	8.68±4.09^⁑^	6.75±3.23^┴₸ ^	0.001

The mean values of serum resistin concentration were statistically significantly different between subjects of all groups (p=0.001). A higher serum resistin concentration was confirmed in the group of patients on antiviral therapy for up to 2 years compared to the control group (8.68±4.09 vs 4.47±1.61 ng/mL, p=0.001). In the group of patients who were on antiviral therapy for more than 2 years, the level of serum resistin was significantly lower compared to the group of patients on antiviral therapy for up to 2 years (6.75±3.23 vs 8.68±4.09 ng/mL, p=0.031). Table 2 shows a significant difference between T2 and the control group (6.75±3.23 vs 4.47±1.61 ng/mL, p=0.007).

### Relationship of serum adiponectin and resistin levels to the liver fibrosis parameters

**Figure 1:**
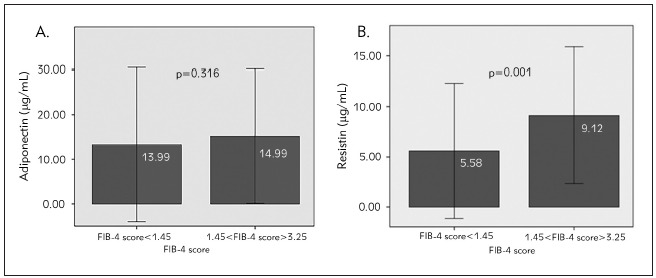
Serum levels of adipokines (A. adiponectin; B. resistin) according to FIB-4 score values in patients with CHB on antiviral therapy Bar chart express the serum adiponectin (Figure 1A) and resistin levels (Figure 1B) in the in chronic hepatitis B virus patients on antiviral therapy with different degree of liver fibrosis according to the values of FIB-4 score (<1.45, 1.45–3.25). The top of the bar represents mean value and the error bars represent standard deviation (±2SD); p<0.001 significant difference

There was no significant difference between mean serum adiponectin levels in patients with a higher FIB-4 score (1.45–3.25) compared to a score lower than 1.45 (15.14±7.50 mg/mL vs 13.25±8.60 mg/mL, p=0.316), Figure 1A. Additionally, the mean serum resistin level was significantly higher in the group of patients with a higher FIB-4 score (9.12±3.39 ng/mL vs 5.58±3.36 ng/mL, p=0.001), Figure 1B.

**Figure 2:**
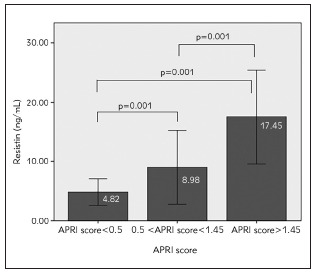
Serum resistin levels according to the APRI score values in patients with CHB Bar charts of serum resistin levels (mg/mL) in the CHB patients with APRI score of different degree of liver fibrosis (<0.5, 0.5–1.45 and >1.45). The top of the bar represents mean value and the error bars represent standard deviation (±2SD); p<0.001 significant difference in mean serum resistin levels between the different degrees of liver fibrosis according to the values of APRI score

Serum adiponectin concentrations in patients with APRI score <0.5 (12.87±8.73 mg/mL), APRI score 0.5-1.45 (15.50±7.36 mg/mL) and APRI score >1.45 (13.09±8.64) did not differ significantly (p=0.378). As opposite of that, there was a significant difference between serum resistin levels depending on the degree of liver fibrosis, according to the values of APRI score (4.82±1.11 ng/mL in APRI score <0.5 vs 8.98±3.08 ng/mL in APRI score 0.5-1.45 vs 17.45±3.96 in APRI score >1.45, p=0.001). The post-hoc Scheffe test confirmed a significant difference in the mean serum resistin levels between each pair of groups (p=0.001), Figure 2.

A significant positive correlation was found between serum concentrations of resistin and liver fibrosis estimated based on the values of FIB-4 (r=0.741, p=0.001) and APRI score (r=0.839, p=0.001). The higher values of serum resistin concentration are associated with higher values of fibrosis score (Table 3). Serum adiponectin levels did not significantly correlate with FIB-4 and APRI score of liver fibrosis in CHB patients, regardless of the duration of antiviral treatment (p>0.05).

**Table 3:** Correlation of serum adiponectin and resistin levels with FIB-4 and APRI score of liver fibrosis in CHB patients on antiviral treatment T1 – CHB patients on antiviral therapy for up to 2 years; T2 – CHB patients on antiviral therapy for more than 2 years

	T1 group				T2 group			
	FIB-4 score		APRI score		FIB-4 score		APRI score	
	rho	p	rho	p	rho	p	rho	p
Adiponectin (μg/mL)	0.152	0.368	0.088	0.604	0.131	0.435	0.139	0.406
Resistin (ng/mL)	0.699	0.001	0.860	0.001	0.784	0.001	0.831	0.001

Univariate two-way ANOVA test showed that progression of liver fibrosis estimated by FIB-4 and APRI scores had a significant effect on resistin values (F 17.42, p=0.001; F 40.09, p=0.001), as well as the length of antiviral treatment (F 8.109, p=0.031), (Table 4). Progression of liver fibrosis expressed by values of FIB-4 score and length of treatment interaction had a significant effect on serum resistin values (F 4.835, p=0.048), while APRI score and length of treatment interaction did not have a significant effect on serum resistin levels (F 4.483, p=0.051). These scores and the length of antiviral treatment did not significantly affect the values of serum adiponectin (Table 4).

**Table 4:** Combined effects of severity disease (estimated by FIB-4 or APRI score) and duration of antiviral therapy on serum resistin and adiponectin concentrations F value – the ratio of the mean-square value for the source of variation to the residual mean square; FIB-4 score^*^ length of antiviral treatment – effects of these two factors interaction on values of the dependent variable; APRI score^*^ length of antiviral treatment – effects of these two factors interaction on values of the dependent variable; the significance of results at p<0.05

Dependent variable:	Resistin (ng/mL)		Adiponectin (mg/mL)	
Independent variables	F	p	F	p
FIB-4 score	17.422	0.001	0.755	0.316
length of antiviral treatment	8.109	0.031	0.196	0.659
FIB-4 score^*^ length of antiviral treatment	4.835	0.048	1.634	0.205
APRI score	40.092	0.001	0.940	0.378
APRI score^*^ length of antiviral treatment	4.483	0.051	1.651	0.203

## Discussion

Treatment and control of CHB may become complicated due to the poor awareness of the disease and lack of screening programs and adequately monitoring of complications [15]. Considering that 15-40% of patients with CHB will progress to cirrhosis and hepatocellular carcinoma (HCC), even with sustained viral suppression during therapy, the use of appropriate serum biomarkers as indicators of disease progression is crucial. Durazzo et al. [16] pointed out the role of serum adiponectin and resistin in the course of CHB and verified a significant decrease in resistin with non-significant reduction in adiponectin after treatment of CHB. Our study examined the variations of serum adipocytokine values in patients with CHB depending on the length of antiviral therapy to determine if they could be appropriate parameters to assess the degree of liver fibrosis and indirectly on the effectiveness of antiviral therapy in controlling disease progression.

The study results showed that mean serum resistin concentration was significantly higher in patients with CHB on antiviral therapy compared to healthy control. We also found a significantly higher value of serum resistin concentration in the early period of antiviral treatment of CHB patients, and the two-way analysis of variance (ANOVA) showed that the combined effects of disease severity assessed by FIB-4 score and duration of antiviral therapy had a significant effect on serum resistin values. Our results are in accordance with the study of Meng et al. [17], which showed higher values of resistin in patients with advanced intrahepatic inflammation and higher stage of fibrosis in three groups of patients-patients with CHB, patients with liver cirrhosis and patients with liver failure as a consequence of CHB. Significantly higher serum resistin levels in that study were found in the latter two groups, considering resistin as a prognostic factor in disease severity. Contrary to these results, the study of Tsochatzis et al. [18] showed that serum resistin levels were lower in patients with severe liver fibrosis, as well as that resistin levels are independently associated with fibrosis severity in patients with chronic hepatitis B and C infection, but it remained unclear to the authors whether lower levels of resistin in advanced fibrosis represent a marker of disease severity or whether resistin is directly implicated in disease progression.

Our results indicated a decrease in adiponectin values in patients with CHB compared to healthy controls, with verified statistically significant difference only between the group of patients on longer antiviral therapy (for more than two years) and the control group. We did not find a significant difference in adiponectin values in patients with CHB on antiviral therapy according to the different degree of liver fibrosis, based on the results of FIB-4 and APRI score, although some discrepancies were observed in the ratio of serum adiponectin levels to higher degrees of liver fibrosis assessed based on these scores. Higher values of adiponectin were observed with a FIB-4 score greater than 1.45. Additionally, in 43 patients with APRI score values between 0.5 and 1.45, serum adiponectin levels showed a tendency to increase insignificantly compared to 29 patients with APRI score lower than 0.5, while in the group of 3 patients with APRI score greater than 1.45, these values were lower. If we take into consideration that only three patients had an APRI score greater than 1.45, the difference found can be taken with caution, and it can be concluded that an increase in adiponectin levels might indicate a higher degree of fibrosis according to the APRI score. These results are partially in accordance with results of some recently published studies, which have shown higher levels of adiponectin in CHB patients with advanced liver fibrosis and inflammation [19] [20], although the question of its elevated values in advanced liver disease remains open because adiponectin is an anti-inflammatory factor that contributes to the reduction of inflammation in the liver parenchyma [21]. A possible explanation for higher adiponectin values in patients with CHB compared to healthy controls lies in the fact that adiponectin is a potent inhibitor of hepatic stellate cells activation, and consequently, its deficiency leads to greater fibrosis [22]. The study investigating the adiponectin-FGF 15/19 axis as an essential axis in the development of alcoholic steatohepatitis determined that ethanol dysregulates adiponectin production, reduces hepatic adiponectin receptors disrupts adiponectin signalling [23]. A higher degree of steatohepatitis is consequently associated with lower levels of adiponectin. Our study showed that the concentration of serum resistin in patients with chronic hepatitis B corresponds to the severity of liver fibrosis and depends on the duration of antiviral therapy.

## Conclusions

This study observed a significantly higher serum resistin level in patients with CHB on the different duration of antiviral therapy compared to healthy control subjects. A higher degree of liver fibrosis in patients with viral hepatitis B was associated with significantly higher serum resistin concentrations. These results suggest that the serum resistin could be a potential non-invasive biomarker of liver fibrosis and its severity in the patients with hepatitis B infection and an indicator of the effects of antiviral therapy on liver histology.

There was no significant difference between the mean serum adiponectin levels in patients with CHB on antiviral therapy concerning the degree of liver fibrosis determined using the FIB-4 and APRI score. Therefore, the role of adiponectin in CHB infection needs to be further studied.

## Dodatak

### Conflict of interest statement

The authors stated that they have no conflicts of interest regarding the publication of this article.
